# Cytokine Release in HR-HPV(+) Women without and with Cervical Dysplasia (CIN II and III) or Carcinoma, Compared with HR-HPV(−) Controls

**DOI:** 10.1155/2007/24147

**Published:** 2007-12-13

**Authors:** Aagje G. Bais, Ilse Beckmann, Patricia C. Ewing, Marinus J. C. Eijkemans, Chris J. L. M. Meijer, Peter J. F. Snijders, Theo J. M. Helmerhorst

**Affiliations:** ^1^Department of Obstetrics and Gynaecology, Erasmus University Medical Center, 3000 CA, Rotterdam, The Netherlands; ^2^Department of Pathology, Erasmus University Medical Center, 3000 CA, Rotterdam, The Netherlands; ^3^Department of Public Health, Erasmus University Medical Center, 3000 CA, Rotterdam, The Netherlands; ^4^Department of Pathology, Vrije Universiteit Medical Center, 1007 MB, Amsterdam, The Netherlands

## Abstract

*Aims*. We investigated the effect of HR-HPV infection on the capacity of the cytokine network in whole blood cultures during carcinogenesis of cervical carcinoma. *Methods*. Thirty-nine women with moderate dysplasia, severe dysplasia, cervical carcinoma, or without dysplasia formed the study group. The control group consisted of 10 HR-HPV-negative women without CIN. Whole blood cultures were stimulated with phytohemagglutinin (PHA) and concentrations of tumour necrosis factor α (TNFα), interferon γ (IFNγ), interleukin 2 (IL-2), interleukin 12 (IL-12), interleukin 4 (IL-4), and interleukin 10 (IL-10) were determined by ELISAs. *Results*. A significant increase in cytokine release was detected in HR-HPV-positive women without dysplasia. In women with cervical cancer, release of IFNγ and IL-12 was of the same magnitude as in HR-HPV-positive women without clinical manifestations. Most Th1-type/Th2-type ratios decreased form CIN II to CIN III, and increased from CIN III to invasive carcinoma. *Conclusions*. (1) Infection with HR-HPV without expression of cervical dysplasia induces activation of the cytokine network. (2) Increases in ratios of Th1-type to Th2-type cytokines at the stage of cervical carcinoma were found by comparison with stage CIN III. (3) Significant changes in the kinetics of cytokine release to a Th2-type immune response in blood of women with cervical dysplasia occurred progressively from CIN II to CIN III.

## 1. INTRODUCTION

It is well established that high-risk (HR) human papillomavirus (HPV) types are causative for the development of cervical cancer [1–3]. The majority of HPV infections are cleared without further consequences for the host, but some infections with HR-HPV types may give rise to high-grade cervical intraepithelial neoplasia (CIN III) and cervical cancer [4–6]. There is evidence that cell-mediated immune responses of the host, both systemic and local, are important determinants for the course of the infection [7]. Cell-mediated immune responses are regulated by T lymphocytes [T-helper (Th) lymphocytes and cytotoxic lymphocytes (CTLs)] in cooperation with antigen-presenting cells (APCs) [monocytes (MCs) and dendritic cells (DCs)]. These cells all release cytokines that can influence one another's synthesis and actions in the setting of an immuno-regulating cytokine network. Cytokines in immune responses to infection are often classified as immuno-stimulating (tumour-suppressing) Th1–type cytokines and immuno-inhibitory (tumour-promoting) Th2-type cytokines. Th1-type cytokines such as interferon γ (IFNγ), tumour necrosis factor α (TNFα), interleukin 2 (IL-2), and IL-12 are produced mainly by lymphocytes, APCs, and natural killer cells (NK-cells). They induce and exhibit cell-mediated immunity. Th2-type cytokines (IL-4, IL-5, IL-6, IL-8, IL-10), produced by lymphocytes and MCs, are immuno-inhibitory for cell-mediated responses and predominantly induce humoral immunity [8, 9]. Qualitative and quantitative analyses of cytokine profiles have been used to characterize the immune response in HPV-related CIN. These were performed with peripheral blood mononuclear cells (PBMCs) [10–12] or with T-cell fractions isolated from PBMCs [13–16] and occasionally with whole blood cultures [17] after stimulation with several antigens. Selective cytokines, mostly IFNγ [11, 12, 14–18], IL-2 [10–14]; and occasionally the APC-derived IL-12 [17] or TNFα [14] were measured together with one or two of the typical Th2-type cytokines IL-4, IL-5, and IL-10 [11, 12, 16, 17]. Generally a shift from a Th1-type to a Th2-type cytokine response was observed when healthy controls or women with low-grade squamous intraepithelial lesions (LSIL) were compared with cases of high-grade SIL (HSIL) or cervical carcinoma [7, 11, 17, 19].

We previously observed manifestation of a Th2-type cytokine pattern in plasma of HR-HPV-positive women during carcinogenesis of cervical cancer at the stage of CIN III [20]. Recent studies with isolated T-cell fractions stimulated with HPV16-derived oncopeptides indicate a reactivation of an inflammatory response in patients with carcinoma [12, 15]. These results let us assume that significant changes in the immunocompetence of circulating leukocytes are involved in the development from cervical dysplasia to cervical cancer.

In the present study we used whole blood cultures from HR-HPV-negative controls, HR-HPV-positive women without cervical dysplasia and HR-HPV positive patients with different grades of CIN and cervical cancer to investigate changes in immunocompetence expressed in the capacity of circulating leukocytes to release cytokines in response to a mitogenic challenge. Of interest were the effect of HR-HPV infection without clinical manifestations, the special position of CIN III with a Th2-type cytokine response, and a possible revival of inflammatory cytokine activity in cervical carcinoma.

## 2. MATERIALS AND METHODS

### 2.1. Patients and controls

Inclusion took place at the outpatient clinic of the Obstetrics and Gynaecology Department of the Erasmus University Medical Center (Rotterdam, The Netherlands) between July 2000 and August 2002. Our selection of patients for this study was based on the presence of HR-HPV and the grade of cervical intraepithelial neoplasia. HPV sampling and a cervical biopsy were carried out on all participating women. Histology results were defined as no dysplasia, mild dysplasia (CIN I), moderate dysplasia (CIN II), severe dysplasia (CIN III), or (micro-) invasive cancer. An experienced pathologist revised all histological samples. Women with CIN I lesions (mild dysplasia) were excluded since more than fifty percent of our patients with CIN I turned out to be HR-HPV-negative. Healthy women who attended the outpatient clinic for a regular sterilisation procedure were recruited as HR-HPV-negative controls after sampling for histology and HPV. Exclusion criteria for all participants were (anamnestic required): postmenopausal state, pregnancy at time of sampling, chronic diseases (diabetes, allergy, auto-immune), presence of sexually transmitted diseases (STDs) and infection with human immunodeficiency virus (HIV), signs of acute infection at time of sampling, and an immune-compromised state. With the exception of oral contraceptives, no participant used medication on a regular base. No participant had used pain-medication (including NSAIDs) for at least two weeks prior to sampling in order to avoid the well-known influence of NSAIDs on cytokine release from PBMCs. The study protocol was approved by the Ethics Committee of the Erasmus Medical Center and all women voluntarily gave signed informed consent.

### 2.2. HPV-sampling and determinations

Cervical scrapes for HPV detection and typing were taken using a cervical bio-sampler (Accellon Combi Medscand Medical, Malmö, Sweden). HPV testing was performed with the consensus GP5+/GP6+ PCR enzyme immunoassay (EIA) using a cocktail probe covering 37 (sub- )types, including all (probably) HR-HPV types, as previously described [21]. This test is clinically validated [22]. We used β-globin PCR to identify sampling errors and to monitor for PCR inhibitors. Additionally, reverse line blot (RBL) analysis was performed on PCR-EIA-positive cases to identify individual HPV types.

### 2.3. Blood sampling

For the preparation of whole blood cultures, peripheral venous blood samples were collected between 8–12 am in sterile endotoxin-free vacutainers (Endo Tubes Chromogenix AB, Mőlndal, Sweden) coated with Na-heparin as anticoagulant, and immediately processed.

For a leukocyte count peripheral venous blood samples, collected between 8 and 12 am, were drawn into endotoxin-free vacutainers (Becton-Dickinson, Meylan, NJ, USA) with ethylene-diaminetetra-acetic acid (EDTA) as anticoagulant and leukocyte counts performed with a Sysmex XE-2100.

### 2.4. Whole blood cultures

For preparation of whole blood cultures, blood was diluted 1:10 with RPMI 1640 culture medium with 25 mM Hepes, supplemented with 10 U/ml penicillin, 100 μg/ml streptomycin, and 4 mM l-glutamine (medium and supplements from Life Technologies BV, Breda, The Netherlands). Diluted blood was distributed in cell culture plates and incubated with phytohemagglutinin (PHA) (Sigma-Aldrish, Mo, USA) dissolved in RPMI medium to a final concentration of 10 μg/ml blood culture, for 96 hours at 37°C and 5% CO_2_. Blood cultures without PHA were run as controls. All cultures were sampled at 0, 24, 48, 72, and 96 hours, centrifuged for 10 minutes at 4°C and 1500 g, and culture supernatants kept at −80°C until analysis.

### 2.5. Cytokine determinations

All samples were analysed by commercially available enzyme-linked immunoassays (Biosource Europe, Nivelle, Belgium) for the cytokines TNFα, IFNγ, IL-2, IL-4, IL-10, and IL-12 [23]. The detecting antibody in the immunoassay for IL-12 recognized the bioactive heterodimeric (p40 + p35) cytokine as well as the subunit p40 monomer or homodimer. According to the manufacturer, the minimal detectable concentrations (MDCs) and intra- and interassay coefficients (CVs) of variation were as follows: TNFα: MDC, 3 pg/ml; CVs, *<*6 and *<*10%; IFNγ: MDC, 2 pg/ml; CVs, *<*5 and *<*10%; IL-2: MDC, 7 pg/ml; VCs, *<*6 and *<*10%; IL-4: MDC, 2 pg/ml; CVs, *<*5 and *<*7%; IL-10: MDC, 1 pg/ml; CVs, *<*5 and *<*10%; IL-12 + p40: MDC, 1.5 pg/ml, CVs, *<*10 and *<*10%.

### 2.6. Statistical analysis

Preliminary Komolgoroff-Smirnov tests showed an abnor mal distribution of cytokine values in PHA-stimulated whole blood cultures. Accordingly, cytokine data are presented as medians with ranges unless stated otherwise. The nonparametric Kruskal-Wallis test (K. W. test) and Mann-Whitney's U-test were used as appropriate to assess differences in cytokine levels between groups. Levels of statistical significance were adjusted for the number of comparisons according to Bonferroni's method, as indicated in the graphics. Differences in patient characteristics between groups were evaluated by one-way ANOVA and unpaired two-tailed T-tests. Spearman's correlations were used to investigate possible relations between age at time of sampling and released cytokines.

## 3. RESULTS

### 3.1. The study groups

Thirty-five patients with different grades of CIN were selected. Five of them were excluded because of diabetes (n=1), allergy (n=2), autoimmune disease (n=1), or acute infection at time of sampling (n=1), leaving 30 women eligible for inclusion: 10 women with moderate dysplasia (CIN II), 10 women with severe dysplasia (CIN III), and 10 women with cervical carcinoma (8 squamous cell carcinoma, 2 adenocarcinoma). All women of this group revealed a positive GP5+/6+ HR-HPV PCR test. We selected 22 healthy women with normal histology. Three of them were excluded because of the presence of allergy (n=2) or acute infection at time of sampling (n=1), leaving 19 healthy women without cervical dysplasia. Nine women had a positive HR-HPV test, 10 women tested negative for HPV-DNA, forming the control group. Baseline characteristics of the study groups are summarized in Table 1.

The mean age of HR-HPV-positive women without cervical dysplasia is significantly lower than in the other groups. This could be expected since first infection without clinical manifestation is frequently observed in young sexually active women. Spearman's correlations between age at time of sampling and released cytokines over the whole group of patients and controls were not significant (data not shown). The changes in immune-competence in our study are not related to age.

### 3.2. The cytokine response

**Figure 1:**
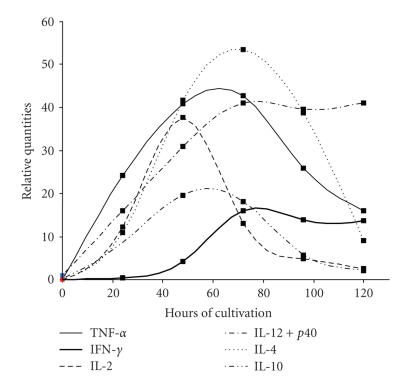
Time course of cytokine release in full blood cultures stimulated with 10 μg PHA per ml.

The results of cytokine assays were calculated per 10^6^ leukocytes, in order to stratify for possible different numbers of cytokine-producing leukocytes between study subjects [20, 24]. Preliminary experiments were carried out on all investigated cytokines to determine the time of peak production in response to PHA stimulation of our whole blood culture system (data not shown). Cytokine concentrations from 0 to 96 hours stimulation time were analysed in at least six randomly chosen study subjects for each stage of CIN. Peak time for TNFα, IFNγ, and IL-12 + p40 production was 72 hours, for IL-2 48 hours of cultivation time. Maximum release for IL-4 and IL-10 varied between 48 and 72 hours. A typical sample for the time-course of cytokine release in our blood culture system is shown in Figure 1.

In general our data of maximum cytokine release are in accordance with kinetic studies of PBMC's [25]. On the basis of these results, IL-2 release was determined after 48 hours, release of TNFα, IFNγ, and IL-12 + p40 after 72 hours; and of IL-4 and IL-10 after 48 and 72 hours of cultivation. For calculations of the latter two cytokines values of maximal release were chosen.

A significant difference in cytokine release was observed between the two groups of women without dysplasia: with the exception of IL-12 all investigated cytokines were significantly increased in HR-HPV-positive women. The results are summarized in Table 2.

**Figure 2:**
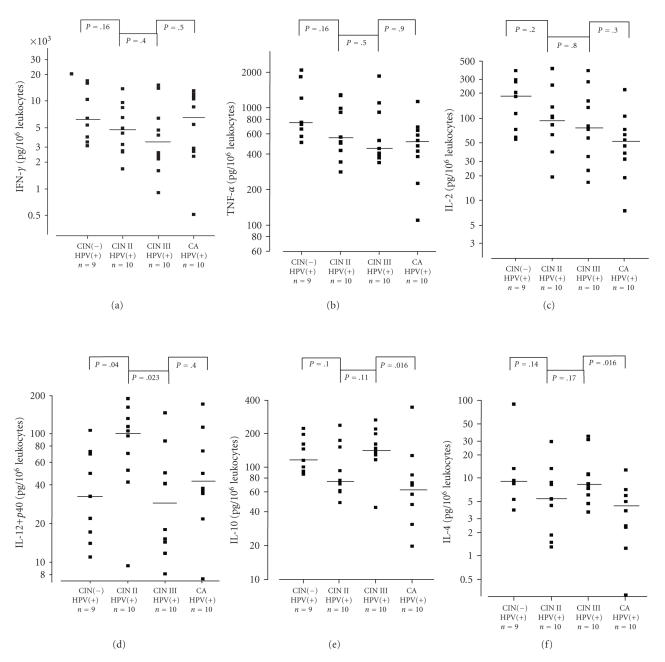
Release of cytokines in full blood cultures of HR-HPV-positive women without and with cervical dysplasia or cervical cancer. Blood cultures were stimulated with 10 μg PHA per ml. Logarithmic scale. A horizontal line indicates median values. Comparisons are significant with P *<*.017. (a): IFNγ, (b): TNFα, (c): IL-2; (d): IL-12 + p40; (e): IL-10; (f): IL-4.

In HR-HPV-infected women, release of Th1-type cytokines IFNγ, TNFα, and IL-2 decreased with increasing grades of CIN. IFNγ increased again from CIN III to carcinoma. IL-12 reached a maximum in CIN II and decreased in CIN III and carcinoma; but the differences between groups were statistically not significant (K. W. test: P=.068 for IL-12 + p40, P=.264 for IFNγ, P=.077 for TNFα and P=.071 for IL-2). The Th2-type cytokines IL-4 and IL-10 behaved differently. Release reached a maximum for IL-10 and IL-4 in patients with CIN III and decreased significantly for both cytokines in patients with invasive carcinoma. (K. W. test: P=.019 for IL-10 and P=.033 for IL-4). The results are summarized in Figure 2.

**Figure 3:**
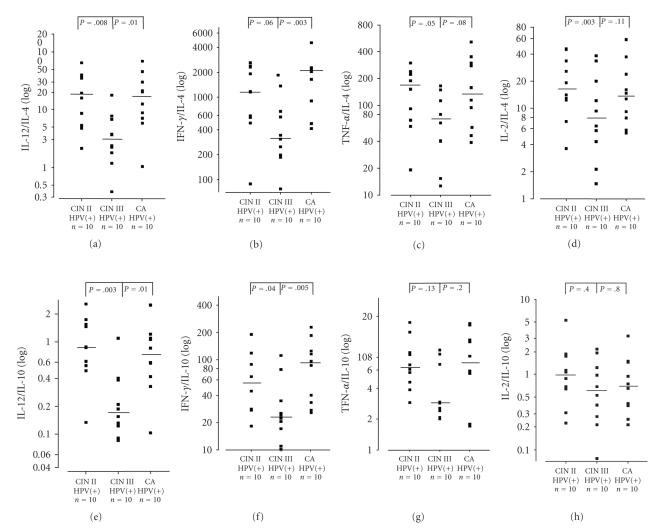
Cytokine ratios in full blood cultures of HR-HPV-positive women with cervical dysplasia and cervical cancer. Blood cultures were stimulated with 10 μg PHA per ml. Logarithmic scale. A horizontal line indicates median values. Comparisons are significant with P *<*.025. (a): IL-12/IL-4; (b): IFNγ/IL-4; (c): TNFα/IL-4; (d): IL-2/IL-4; (e): IL-12/IL-10; (f): IFNγ/IL-10; (g): TNFα/IL-10; (h): IL-2/IL-10.

In order to characterize a possible Th1-type/Th2-type shift we calculated the ratios of Th1-type cytokines IL-12, IFNγ, TNFα, and IL-2 to Th2-type cytokines IL-10 and IL-4 in HR-HPV infected groups. (Results of K. W. tests: IL-12/IL-10 P=.005, IL-12/IL-4 P=.01, IFNγ/IL-10 P=.013, IFNγ/IL-4 P=.015, TNFα/IL-10 P=.303, TNFα/IL-4 P=.096, IL-2/IL-10 P=.642, IL-2/IL-4 P=.251). There was a significant decrease in Th1-type/Th2-type ratios between CIN II and CIN III for IL-12/IL-4 and IL-12/IL-10. Also, IFNγ/IL-4 and IFNγ/IL-10 showed a similar though statistically not significant trend as demonstrated in Figure 3. Values increased again in invasive carcinoma compared to CIN III. This increase was significant for IL-12/IL-4, IL-12/IL-10, IFNγ/IL-4, and IFNγ/IL-10.

In order to characterize a possible Th-1 type cytokine pattern after establishment of an invasive carcinoma we compared cytokine levels in PHA-stimulated blood cultures of patients with invasive carcinoma with levels in HR-HPV-positive women without dysplasia. There was no difference between levels of IL-12 + p40 and IFNγ in both groups, but release of TNFα and IL-2 as well as of IL-10 and IL-4 was significantly lower in patients with carcinoma. The results are summarized in Table 3.

## 4. DISCUSSION

The significant increase in Th1-type as well as Th2–type cytokines in our HR-HPV-positive women with normal histology suggests viral activation of the systemic cytokine network and induction of cell-mediated immunity after initial HR-HPV infection (Table 2). To our knowledge this is the first description of activation of the systemic cytokine network in HR-HPV-positive women without dysplasia.

Cytokine release changed to an antiinflammatory, tumour-promoting pattern by increase in IL-4 and IL-10 expression at the stage of CIN III. This result confirms and extends our earlier observations of a change to a Th2-type cytokine pattern in the circulation of patients with CIN III [20] and is in agreement with earlier studies showing a shift from Th1-type to Th2-type cytokines during carcinogenesis. Clerici et al. [11] observed decreased IFNγ and IL-2 and increased IL-4 and IL-10 in mitogen-stimulated cultures of PBMCs isolated from women with CIN III when compared with cultures from HR-HPV-negative women. Jacobs et al. [17] described increased IL-10 and decreased IL-12 release in whole blood cultures of patients with HSIL when compared with HR-HPV-negative controls. Tsukui et al. [10] stimulated PBMCs of patients with cervical dysplasia and carcinoma with HPV-16 peptides. They found decreasing IL-2 release with increasing severity of the disease, which is in agreement with our results for IL-2. The observed minimium for IFNγ release in CIN III but not in invasive carcinoma differs from the observations of an earlier study by Mori et al. [18] where PHA-stimulated IFNγ release from PBMCs in cases of invasive carcinoma was significantly decreased when compared with data from healthy women. In the study of Mori et al. however, the presence of HR-HPV was not investigated, which might explain the difference in results with our study.

A shift to a Th2-type cytokine pattern in CIN III was more obvious when the ratios between Th1-type and Th2-type cytokines (Figure 2) are evaluated. They show a tumour-promoting change in cytokine balance, significant for IL-12/IL-4 and IL-12/IL-10, and a trend for IFNγ/IL-4, IFNγ/IL-10, and TNFα/IL-4. Our study describes for the first time changes in the cytokine pattern within the cytokine network, developing from HR-HPV infection without clinical symptoms via CIN II and CIN III to carcinoma.

The course of IL-12 secretion in our study groups merits consideration. IL-12 is one of the first cytokines released during an innate immune reaction and stimulates a Th-1 type cytokine response in cell-mediated immunity. Our HR-HPV-positive women with normal histology demonstrated significantly increased Th1- and Th2-type cytokine release, with the exception of IL-12 which was low. Our observation of high secretion of IL-12 in CIN II might be explained by an observation made by Moscicki et al. [26]. These authors reported high levels of IL-12 in cervical mucous in HSIL and hypothesized that high IL-12 levels could represent a defence mechanism in turning on a Th1-type antitumour response and, as IL-12 is known to inhibit angiogenesis, preventing growth of a tumour.

The significant increase of the four cytokine ratios between CIN III and carcinoma may indicate that the presence of a tumour with an inflammatory reaction and exposure of viral antigens (high viral load) eventually induces a certain T-cell response. This response remains incomplete as shown in our cytokine data presented in Table 3. Values of IFNγ and IL-12 release in cervical carcinoma are comparable to data obtained after initial HR-HPV infection; all other cytokine levels remain significantly lower. These results suggest a second deregulated and incompetent immune response in cervical carcinoma, probably due to manifestation of an inflammatory effect of the tumour itself. This reaction is partly comparable to the inflammatory reaction on the initial HR-HPV infection, as expressed in the ratio's of IFNγ and IL-12 in Figures 3(a), 3(b), 3(e), 3(f). These results are in agreement with observations of de Jong et al. [12], and Steele et al. [15] studied T-cell responses to HPV 16 oncoproteins by measuring IFNγ release in women with low- and high-grade CIN and cervical carcinoma and found higher levels of T-cell responses in carcinoma patients compared to high-grade CIN cases. A similar observation was made by de Jong et al. who investigated HPV16-positive women [12]. This study reports a higher frequency of HPV16-specific CD4+ T-cell responses in patients with cervical carcinoma than in women with CIN III lesions.

The increase in the IFNγ/IL-4 ratio found in our study was not observed by de Jong et al. [12] when T-cell cultures were stimulated with PHA. In part, this discrepancy might be owing to differences in HR-HPV types within the study groups since de Jong et al. only selected patients infected with HPV 16. Correlations between specific HPV types and IFNγ release, possibly influenced by IFNγ gene polymorphisms, are suspected but not yet fully investigated [27].

It was our goal to study changes in the cytokine network in blood of HR-HPV-infected women at various stages of CIN upto onset of cervical carcinoma. The cytokine network is probably best represented in a whole blood culture. The use of whole blood cultures for determination of mitogen-stimulated cytokine release by immunocompetent leukocytes has distinct advantages over cultures of isolated leukocytes or lymphocytes. It permits interaction between different leukocytes, preserves concentrations of stimulatory and inhibitory mediators, and avoids activation and changes in cell ratios associated with procedures of isolation and purification [23]. For stimulation of the cytokine network we chose the mitogen PHA. PHA activates mainly lymphocytes and induces rapid cell proliferation together with release of inflammatory and immune cytokines. Endotoxin (LPS) as used in Jacobs' study [17] induces mainly inflammatory cytokines but almost no lymphocyte-derived interleukins [28].

Most studies dealing with cytokine patterns in HR-HPV-related cervical neoplasia and cancer concentrate on infections with HPV 16 (the most frequently observed oncogenic HPV type in Caucasian population) [4]. In contrast to these studies we did not select our patients for particular HR-HPV types. The small sample size of our study groups did not allow us to correlate cytokine response with specific HR-HPV-types. Further studies with enlarged numbers of participants are needed to investigate the individual impact of different HR-HPV-types on the cytokine network.

## 5. CONCLUSIONS

1. Our study suggests that infection with HR-HPV in women without cervical dysplasia induces activation of the cytokine network.

2. Manifestation of a tumour induces a second deregulated and incompetent immune response.

3. Our results confirm and expand our earlier observations on circulating cytokines: significant changes in the kinetics of cytokine release to a Th2-type immune response in blood of women with cervical dysplasia occur progressively from CIN II to CIN III.

These immunological findings are supported by clinical observations: many CIN I or II lesions usually regress without treatment, whereas CIN III lesions mostly will develop into invasive cancer if not properly treated [29].

## Figures and Tables

**Table 1:** Baseline characteristics of study groups.

	No CIN	No CIN	CIN II	CIN III	CA	Statistical significance
	HPV neg	HPV pos				
	n=10	n=9	n=10	n=10	n=10	
Age at time of sampling*	36.9	27.4	32.5	31.9	34.6	P=.019
	(6.1)	(6.9)	(5.4)	(4.5)	(6.9)	—
STD in history	0/10	3/9	4/10	3/10	1/10	P=.172
Use of OCs	5/10	6/9	5/10	6/10	7/10	P=.139
Smoking at time of sampling	3/10	5/9	6/10	9/10	4/10	P=.073

HR-HPV types (n)						
16	—	0	5	7	2	—
18	—	0	2	1	5	—
31	—	4	2	2	0	—
other	—	10	4	3	3	—
multiple infections	—	3	2	3	0	—

*years; mean (standard deviation), one-way ANOVA STD = sexually transmitted disease OCs = oral anticontraceptives.

**Table 2:** Influence of HR-HPV infection on the cytokine network in women without cervical dysplasia.

GROUP	N	IL-12 + p40*	IFN-γ*	TNF-α*	IL-2*	IL-10*	IL-4*
HR-HPV(−)	10	25	2406	433	32	58	3
	—	(13–170)	(364–10795)	(68–1714)	(3–192)	(11–140)	(0.4–14)
HR-HPV(+)	9	32	6378	723	183	115	9
	—	(11–106)	(3064–30452)	(504–2244)	(55–378)	(86–221)	(4–89)

Statistical significance	P=	.87	.001	.006	.009	.027	.034

*pg/10^6^ leukocytes, values median (range).

**Table 3:** Cytokine levels in PHA-stimulated blood cultures of CIN(−) women with HR-HPV infection and women with invasive cervical carcinoma.

GROUP	N	IL-12 + p40*	IFNγ*	TNFα*	IL-2*	IL-10*	IL-4*
CIN(−)	9	32	6378	723	183	115	9
HR-HPV(+)	—	(11–106)	(3064–30452)	(504–2244)	(55–378)	(86–221)	(4–89)
Carcinoma	10	43	6975	497	50	63	4
	—	(7–170)	(509–13004)	(110–1127)	(8–220)	(20–342)	(0.3–13)

Statistical significance	P=	.37	.33	.011	.009	.011	.011

*pg/10^6^ leukocytes, values median (range).
